# Improving the Management of Neuropsychiatric Presentations in Early Intervention Services (EIS)

**DOI:** 10.1192/bjo.2024.364

**Published:** 2024-08-01

**Authors:** Henry Finn, Emily Easter

**Affiliations:** South West London and St George's Mental Health Trust, London, United Kingdom

## Abstract

**Aims:**

Early Intervention Services (EIS) are in a unique position to assess patients presenting with their first episode of psychosis. The possibility that an organic disorder may be underlying their presentation must be ruled out, often necessitating neuroimaging and/or input from neurology and neuropsychiatry.

We aim to improve the management of neuropsychiatric presentations in EIS. We will determine the incidence of cases, from the London Boroughs of Sutton and Merton, which warrant referral to neurology, neuropsychiatry and neuroimaging. We will then review referral pathways and provide justification for community services, such as EIS, to *autonomously* request referrals and neuroimaging.

**Methods:**

We retrospectively reviewed the complete caseloads of EIS for Sutton and Merton (n = 121). We considered the neurological comorbidities of patients to determine the incidence of cases which warranted a referral to neurology, neuropsychiatry and/or neuroimaging. We reviewed how requests were made and the subsequent results.

**Results:**

15% of the EIS caseload had a neurological comorbidity. Migraine was the most common condition (8.3%), followed by traumatic brain injury (3.3%), headache (2.5%), and seizure (1.7%). There was one case each of epilepsy, stroke, transient ischaemic attack, cavernoma and cerebral venous thrombosis. 83% of patients with a neurological comorbidity had received neuroimaging and all imaging results were either normal or confirmed known pre-existing neurological disease. The 17% of patients who did not receive neuroimaging had only migraine as a neurological comorbidity. One patient was reviewed by neurology and diagnosed with psychosis presumed to be secondary to paraneoplastic syndrome. All patients that fulfilled criteria for a neuropsychiatry referral had this completed electronically. However, there was no clear pathway to request a review by neurology, and Sutton EIS had difficulties autonomously requesting and accessing the results of neuroimaging, delaying provision of appropriate care.

**Conclusion:**

There is a small but significant burden of neurological comorbidity among EIS patients. In our brief study, we found one patient whose symptoms of psychosis were likely attributable to an organic cause. Accessible pathways to refer patients for neuroimaging, and subsequently to neurology and/or neuropsychiatry if indicated, are crucial in the assessment and management of first episode psychosis where an organic cause is suspected. Access to these resources should be efficient and autonomous for EIS. We are in the process of implementing referral guidance alongside a direct electronic referral process to request neuroimaging and further input from neurology and/or neuropsychiatry, to optimise care for patients and our service.

